# Clinical practice guidelines for the treatment of squamous cell carcinoma of the head and neck: an assessment based on the AGREE II, AGREE-REX tools and the RIGHT checklist

**DOI:** 10.3389/fonc.2024.1442657

**Published:** 2024-12-18

**Authors:** Yingzhen Zhang, Junjie Liu, Shuyu Liu, Ying Zhang, Xingzhou Su, Shaohui Huang, Weiyi Duan

**Affiliations:** ^1^ Department of Oral Maxillofacial-Head and Neck Surgery, School and Hospital of Stomatology, China Medical University, Shenyang, Liaoning, China; ^2^ School and Hospital of Stomatology, China Medical University, Liaoning Province Key Laboratory of Oral Diseases, Shenyang, Liaoning, China

**Keywords:** squamous cell carcinoma, head and neck tumor, AGREE II, AGREE-REX, RIGHT, clinical practice guidelines

## Abstract

**Objective:**

The aim of this study was to obtain several published clinical practice guidelines (CPGs) for Head and neck squamous cell carcinoma (HNSCC) and critically assess and compare their quality by using three guideline quality evaluation tools, namely, AGREE II, AGREE-REX, and RIGHT, to support the development of future CPGs for HNSCC.

**Methods:**

Clinical practice guidelines related to the diagnosis and treatment of HNSCC were screened through a comprehensive systematic literature search. Data were extracted from the guidelines which met the inclusion criteria, and two experienced head and neck oncology surgeons were trained to act as independent reviewers. The quality of the retrieved guidelines that met the inclusion and exclusion criteria was evaluated by using the AGREE II, AGREE-REX, and RIGHT tools. Then, the quality of the guidelines that met the criteria was assessed. Finally, conclusions and recommendations were drawn based on the scoring results.

**Results:**

A total of eight guidelines met the inclusion criteria. Four guidelines(written by ASCO, NCCN, CCO and KCE) scored > 60% in five or more AGREE II quality domains, two guidelines(written by ASCO and KCE) scored > 60% in all AGREE-REX quality domains, and two guidelines(written by ASCO and KCE) scored > 60% in all quality domains on the RIGHT checklist and were considered “recommendable”.

**Conclusions:**

The authors recommend consulting the American Society of Clinical Oncology guidelines for HNSCC and suggest that future guideline development groups refer to the guideline evaluation framework for guideline writing to enhance the applicability and effectiveness of clinical practice guidelines.

## 1 Introduction

Squamous cell carcinoma of the head and neck is a malignant tumor that occurs on the mucosal surfaces of the upper respiratory and digestive tracts (paranasal sinuses, nasopharynx, oropharynx, larynx, oral cavity, and nasal cavity). It accounts for approximately 90% of malignant tumors of the oral cavity and is the sixth most common malignant tumor in the world, with an annual incidence of approximately 0.7 million in 2020 and estimated annual mortality of approximately 350,000 (1). Because nasopharyngeal carcinoma is another uniquely characterized disease, with its epidemiology, clinical presentations, pathological manifestations, and treatments different from those of other squamous cell carcinomas of the head and neck, its clinical practice guidelines were excluded from this evaluation.

A clinical practice guideline (CPG) is defined as a guideline or recommendation that is systematically developed to assist clinicians and patients in the appropriate management of a specific clinical situation (2). Given the increasing research and understanding of head and neck squamous cell carcinoma (HNSCC) in recent years, several CPGs for HNSCC have been issued by organizations and committees in different countries, but their reliability has been questioned because of their varying quality (3). Clear and easy-to-understand CPGs are essential for the implementation of medical interventions in clinical practice (4). Therefore, it is essential to assess the quality of relevant guidelines to help improve the prevention of HNSCC and guide the clinical practice of treatment. Although guidelines for HNSCC have been evaluated globally (5, 6), they have all been evaluated at the methodological level based on the AGREE II tool, and no article has yet been published that independently assesses head and neck squamous cell carcinoma clinical practice guidelines using the AGREE-REX and RIGHT tools in terms of reporting quality and clinical applicability of guidelines.

The quality of clinical practice guidelines consists of two main aspects, namely, methodological quality and reporting quality (7, 8), and tools for assessing the quality of guideline reporting should be distinguished from those for assessing their methodology, as they differ in the purpose, structure, and content (9, 10). Among the various tools for evaluating the methodological quality of guidelines, the AGREE II tool has been shown to be valid and has been widely used in different areas of clinical practice. For the quality of guideline reporting, the RIGHT checklist has now been applied in a wide range of applications. It has been argued that these two evaluation tools should be combined to obtain a more comprehensive evaluation of guidelines (11). In this study, a rigorous methodological and reporting quality evaluation of guidelines that were selected by certain criteria was performed by using these two tools to identify areas for improvement in the guidelines. In this context, the main objectives of this study were to i) assess the quality of eligible clinical practice guidelines; ii) analyze the strengths and weaknesses of each guideline; and iii) synthesize the quality of the evidence and the strength of the associated recommendations to arrive at recommended clinical practice guidelines.

## 2 Materials and methods

### 2.1 Literature search and guideline selection

The following keywords were used from October 2023 to January 2024: “head and neck squamous cell carcinoma”, “head and neck neoplasms”, “guidelines”, and “clinical practice guidelines”, and their extensions were subjected to a literature search in PubMed, EMBASE, Web of Science, and Google Scholar to identify clinical practice guidelines for HNSCC. The reference sections of the retrieved papers were also examined for additional included articles. In addition, the websites of various scientific societies and international associations, such as the American Society of Clinical Oncology (ASCO), the European Society of Medical Oncology (ESMO), the Guidelines International Network (GIN), and the National Institute for Health and Care Excellence (NICE), were searched. The gray literature was also searched through ProQuest and Turning Research Into Practice. Throughout the search, guidelines were available in full text in English, with no restrictions on the year of publication, but for guidelines published by the same organization or institution, the most recent version that could be accessed at the time of writing was used.

### 2.2 Inclusion and exclusion criteria

#### 2.2.1 Inclusion criteria

A. Clinical practice guidelines containing “statements” or “guidelines” or providing “recommendations” for HNSCC;

B. Treatment (conceptual) recommendations for patients diagnosed with head and neck squamous cell carcinoma in the oral cavity, oropharynx, and larynx (population) based on the literature and expert opinion;

C. Authored by world-renowned medical association and other bodies;

D. Guidelines with English language only.

#### 2.2.2 Exclusion criteria

A. Clinical practice guidelines (CPGs) without treatment recommendations (for diagnosis, care, referral, etc.);

B. Nonguidelines;

C. Guidelines for squamous cell carcinoma at sites other than the head and neck;

D. Guidelines for other malignant tumors of the head and neck;

E. Other guideline types that are not clinical practice guidelines (e.g., service guidelines);

F. Full text in English is not available;

G. Previous versions of guidelines published by the same organization or association;

H. Guidelines not developed by a widely recognized medical association and other bodies.

### 2.3 Data screening

In the first stage, the retrieved documents were initially screened by reading the titles and abstracts; in the second stage, the full texts of the documents screened in the first step were read and rechecked to determine whether they met the above inclusion and exclusion criteria. Two independent reviewers (YZ, JL) carried out the above tasks to assess the eligibility of the guidelines. If there was a disagreement between the reviewers during the screening process, a joint discussion was held with a third expert reviewer(SL), and the guidelines were rescreened after a consensus was reached.

### 2.4 Data extraction

Two reviewers were independently responsible for extracting and documenting the following information from the included literature: basic information: title, journal name, authoring group/organization, year of publication, country or region of origin, purpose of the study, target population, and source of funding; (2) recommendations: strengths and limitations of the evidence, criteria for eligibility of the evidence, search strategy, content of the guideline, and the key recommendations described; and (3) quality control: whether the content is influenced by funding, conflicts of interest among writing team members and the external review process, guideline updating process, etc., and the design of information extraction forms based on AGREE II, AGREE-REX, and RIGHT entries.

### 2.5 Evaluation tools and scoring criteria

**Figure 1:**
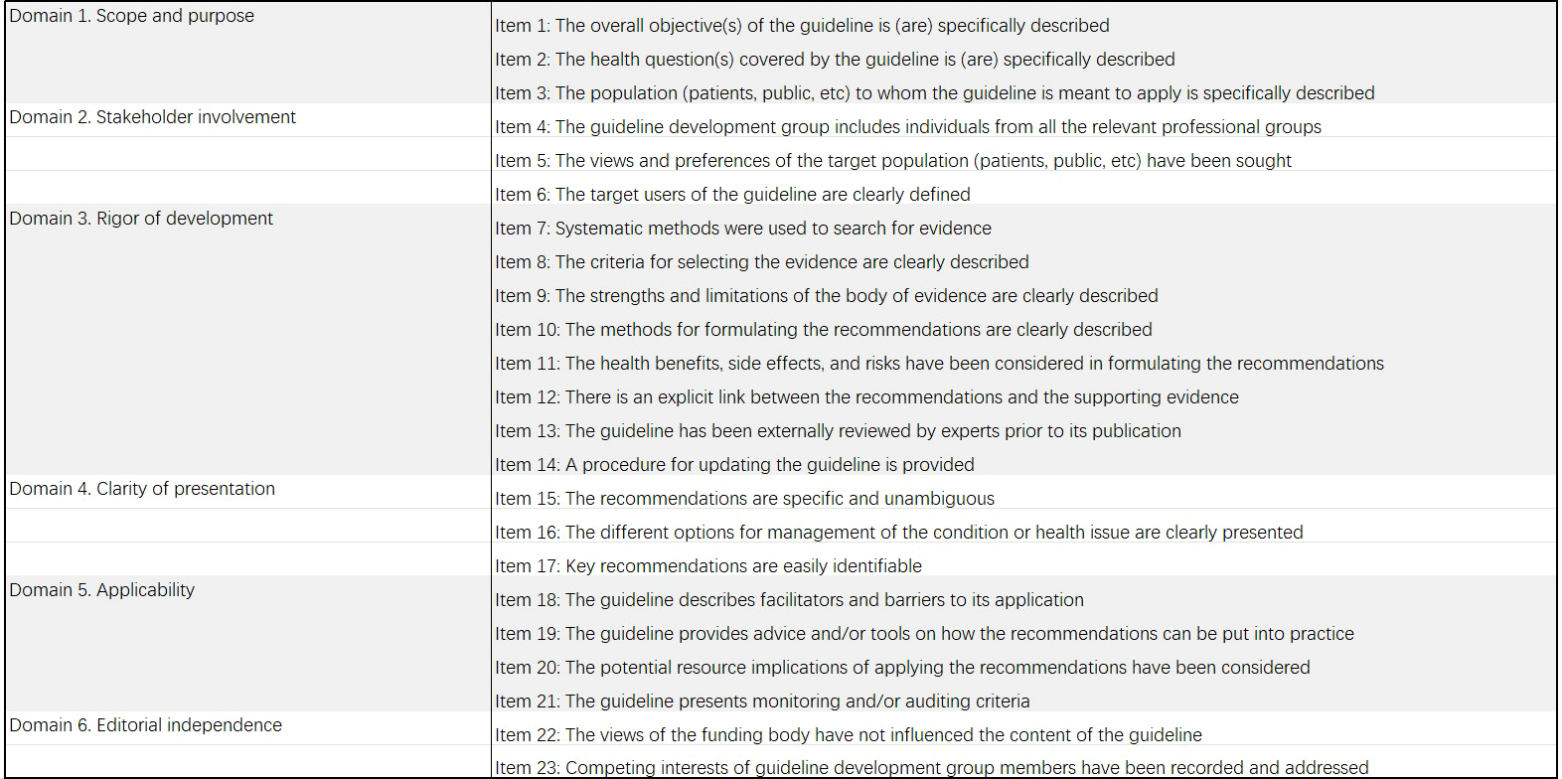
Description of AGREE II domains and items.

**Figure 2:**
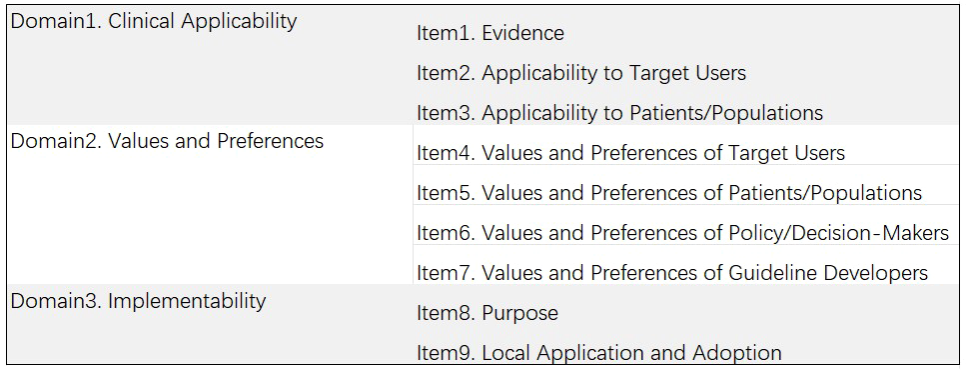
Description of AGREE-REX domains and items.

A. AGREE II and AGREE-REX tools: AGREE II was developed by the international Appraisal of Guidelines for Research and Evaluation (AGREE) team as a methodological tool for research and evaluation of guidelines and has been widely used worldwide (12). Six domains, namely, (1) scope and purpose; (2) stakeholder involvement; (3) rigor of development; (4) clarity of presentation; (5) applicability; and (6) editorial independence, comprising 23 items, were evaluated by two independent reviewers. As the AGREE II tool is not sufficient to ensure that guideline recommendations are credible or implementable when applied to the clinic, the 2019 International Guidelines Research Team further developed the AGREE-REX tool. This tool complements rather than replaces AGREE II (13). AGREE-REX aims to ensure guideline credibility, reliability, and implementability in clinical settings (14) and is organized into the following 3 domains: (1) clinical applicability; (2) values and preferences; and (3) implementability, with 9 items under each of the 3 domains (The specifics of the two tools were illustrated in **Figures 1**, **2**). In this study, after evaluating the selected guidelines using the AGREE II tool, the recommended guidelines were evaluated again using the AGREE-REX tool. Each item was rated by two independent reviewers using a 7-point scale (1-strongly disagree to 7-strongly agree). The score for each domain was calculated as follows: (actual score - minimum possible score)/(maximum possible score - minimum possible score) × 100%, according to the guidance in the AGREE tool manual (15), and 60% was used as the domain pass threshold (16–18). In the use of the AGREE II tool, the guidelines were categorized and rated for quality according to the number of domains with a score of ≥ 60% as follows: ≥ 5 domains were considered “high quality”, 3-4 domains were considered “average quality”, and ≤ 2 domains with a score of ≥ 60% were considered “low quality” (19). Similarly, in the use of the AGREE-REX tool, this study employed a calculation of the percentage of “qualified” domains (i.e., mean domain score ≥ 60%) out of the total number of domains. This calculation yielded four possible values: 0%, 33.3%, 66.7%, and 100%. This is due to the fact that the AGREE-REX tool has only three domains. In this study, 100% of domains with an average score of ≥60% are considered to represent the standard for “high quality”, 66.7% is considered to represent “average quality”, and 0% and 33.3% are considered to represent “low quality”.

**Figure 3:**
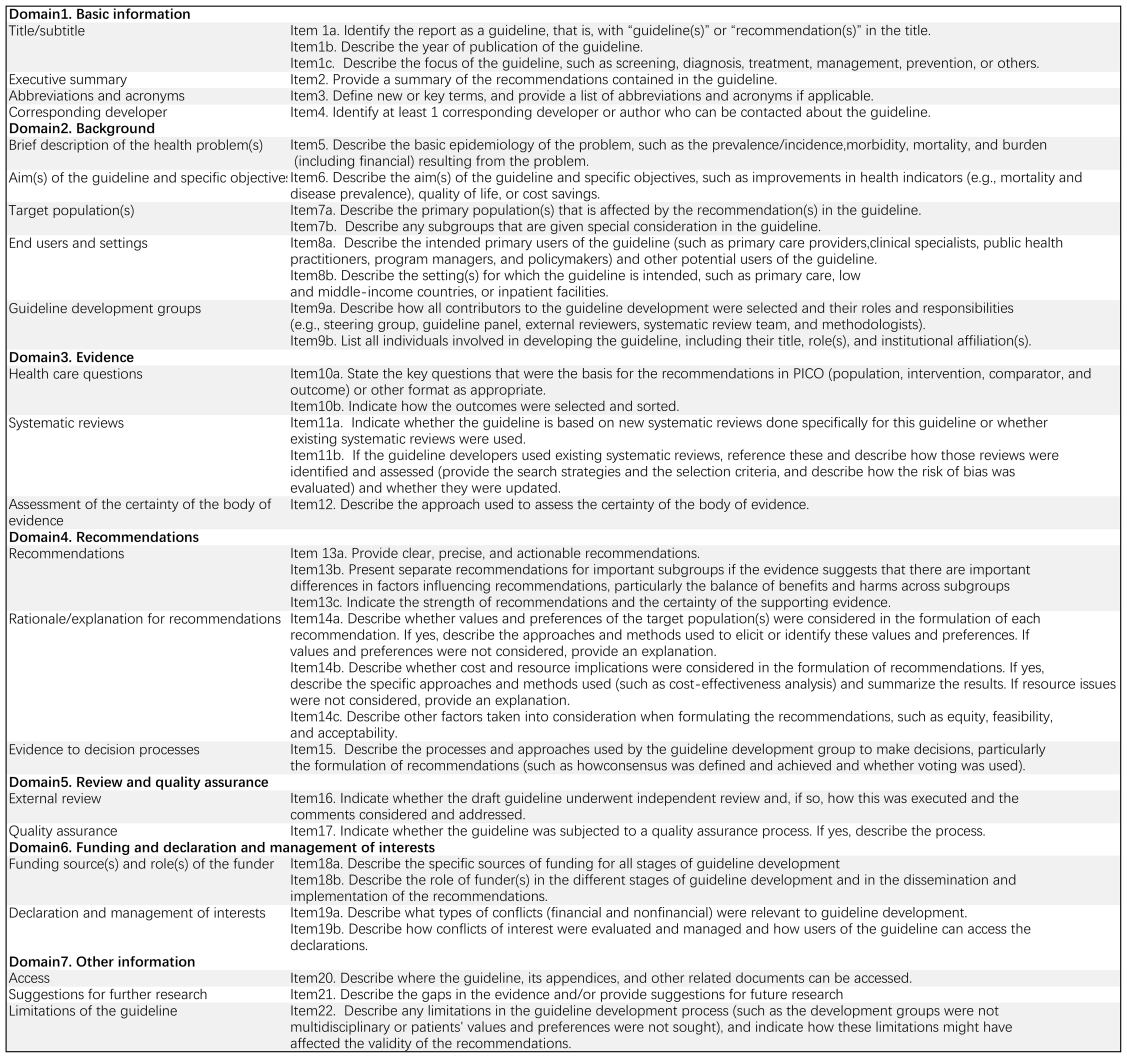
Description of RIGHT domains and items.

B. The RIGHT (Reporting Items for practice Guidelines in Healthcare Checklists): High-quality guidelines need to not only meet rigorous methodological standards but also standardize the reporting format (20). The RIGHT checklist, published by the International RIGHT Working Group in 2016, is a tool for evaluating guidelines from the perspective of guideline reporting standardization (9) and is now widely used internationally. The RIGHT checklist is divided into 7 domains and 22 items as follows: (1) basic information; (2) background; (3) evidence; (4) recommendations; (5) review and quality assurance; (6) funding and declaration and management of interests, and (7) other information (The specifics of the RIGHT tools was illustrated in **Figure 3**). The reporting rate for each guideline item was calculated as follows: items evaluated as “fully reported” were given 2 points, items evaluated as “partially reported” were given 1 point, and items evaluated as “not reported” and “not applicable” were given 0 point. The domain score was calculated as follows: (Interrater score for the domain/highest possible score for the domain) x 100%. This study assessed the quality of the guidelines by calculating the total average score of the average scores across the seven domains. A total average score of ≤ 60% is indicative of “low quality”, 60%-80% is indicative of “average quality”, while > 80% is indicative of “high quality”.

Two independent reviewers calculated the scores for each domain using each of the three tools and subsequently tabulated the results using Microsoft Office Excel 2021 software, as illustrated in **Tables 1**–**3**. The color red represents low scores, green represents high scores, and amber represents moderate scores. Additionally, the darker the red color, the smaller the value, and the darker the green color, the larger the value. In addition, the reviewers were consulted for their recommendations for this study in the context of the corresponding knowledge of this paper or in the context of the reviews provided.

**Table 1:** AGREE II domain scores for the 8 identified HNSCC CPGs.

Guidelines	Domain 1	Domain 2	Domain 3	Domain4	Domain 5	Domain 6	Average score	Quality
	Scope and purpose(%)	Stakeholder involvement(%)	Rigor of development(%)	Clarity of presentation(%)	Applicability(%)	Editorial independence(%)		
EHNS/ESMO/ESTRO	55.6	47.2	32.2	100	68.8	95.8	66.6±27.0	Average
SEOM	80.6	61.1	50	88.9	43.8	87.5	68.7±19.6	Average
ASCO	100	100	96.9	100	100	100	99.5±1.3	High
NCCN	72.2	61.1	63.5	97.2	60.4	12.5	61.2±27.5	High
JAPANESE	83.3	75	57.3	88.9	39.6	87.5	71.9±19.6	Average
KOREAN	75	58.3	70.8	66.7	22.9	50	57.3±19.1	Average
CCO	91.7	80.6	95.8	100	66.7	100	89.1±13.1	High
KCE	91.7	100	97.9	97.2	89.6	91.7	94.7±4.2	High
Mean±SD	81.3±13.9	72.9±19.6	70.6±24.5	92.4±11.4	61.5±25.7	78.1±31.0		

Red indicates Low; amber indicates moderate; and green indicates high.

**Table 2:** AGREE-REX domain scores for the 8 identified HNSCC CPGs.

Guidelines	Domain 1	Domain 2	Domain 3	Average(%)	Domains with
	Clinical applicability (%)	Values and preferences (%)	Implementability (%)		scores≥60%(%)
EHNS/ESMO/ESTRO	52.8	10.4	37.5	33.6±21.5	0
SEOM	55.6	29.2	45.8	43.5±13.3	0
ASCO	97.2	81.3	100	92.8±10.1	100
NCCN	77.8	25	37.5	46.8±27.6	33.3
JAPANESE	83.3	27.1	54.2	54.9±28.1	33.3
KOREAN	77.8	12.5	37.5	42.6±33.0	33.3
CCO	86.1	29.2	54.2	56.5±28.5	33.3
KCE	88.9	87.5	91.7	89.4±2.1	100
Mean±SD	77.4±15.7	37.8±29.7	57.3±24.9		

Red indicates Low; amber indicates moderate; and green indicates high.

**Table 3:** RIGHT domain scores for the 8 identified HNSCC CPGs.

Guidelines	Domain 1	Domain 2	Domain 3	Domain 4	Domain 5	Domain 6	Domain 7	Average	Quality
	Basic information(%)	Background(%)	Evidence(%)	Recommendations(%)	Review and quality assurance(%)	Funding and declaration and management of interests(%)	Other information(%)		
EHNS/ESMO/ESTRO	91.7	56.3	40	39.3	50	43.8	50	53.0±18.1	Low
SEOM	100	75	60	71.4	50	18.8	33.3	58.4±27.2	Low
ASCO	100	93.8	90	100	100	87.5	100	95.9±5.4	High
NCCN	100	65.6	55	50	0	0	83.3	50.6±38.5	Low
JAPANESE	91.7	71.9	75	75	37.5	75	66.7	70.4±16.4	Average
KOREAN	91.7	68.8	75	53.6	37.5	0	50	53.8±29.7	Low
CCO	91.7	90.6	95	67.9	100	50	83.3	82.6±17.7	High
KCE	95.8	90.6	100	96.4	87.5	75	91.7	91.0±8.19	High
Mean±SD	95.3±4.1	76.6±13.7	73.8±21.0	69.2±21.5	57.8±35.3	43.8±34.5	69.8±23.5		

Red indicates Low; amber indicates moderate; and green indicates high.

### 2.6 Quality control

The intraclass correlation coefficient (ICC) is one of the indicators for measuring the reliability of the observer and the reliability of the retest. In this study, we used the SPSS 25.0 software to calculate the ICC for the evaluation results of the two independent reviewers to test the consistency of the evaluations provided by the evaluators. When the ICC is <0.4, the consistency is not good; when the ICC is between 0.4 and 0.59, the consistency is average; when the ICC is between 0.6 and 0.74, the consistency is good; and when the ICC is greater than 0.75, the consistency is high. Prior to the formal start of the evaluation, two reviewers with methodological and professional expertise were systematically trained to ensure consistency in their understanding of the entries. Preevaluations of the guidelines were conducted to calculate the ICCs of the evaluation results. When the ICC was <0.75, the two reviewers discussed the entries with large differences in ratings and reevaluated the entries after reaching a consensus, until the ICC was ≥0.75, at which time the evaluation consistency was considered to be high, and the formal evaluation could begin. After the formal evaluation, the correlation coefficient within the group was recalculated to ensure that the ICC was ≥0.75, and then the data were recorded and analyzed.

## 3 Results

### 3.1 Essential features for inclusion in the guidelines

**Figure 4:**
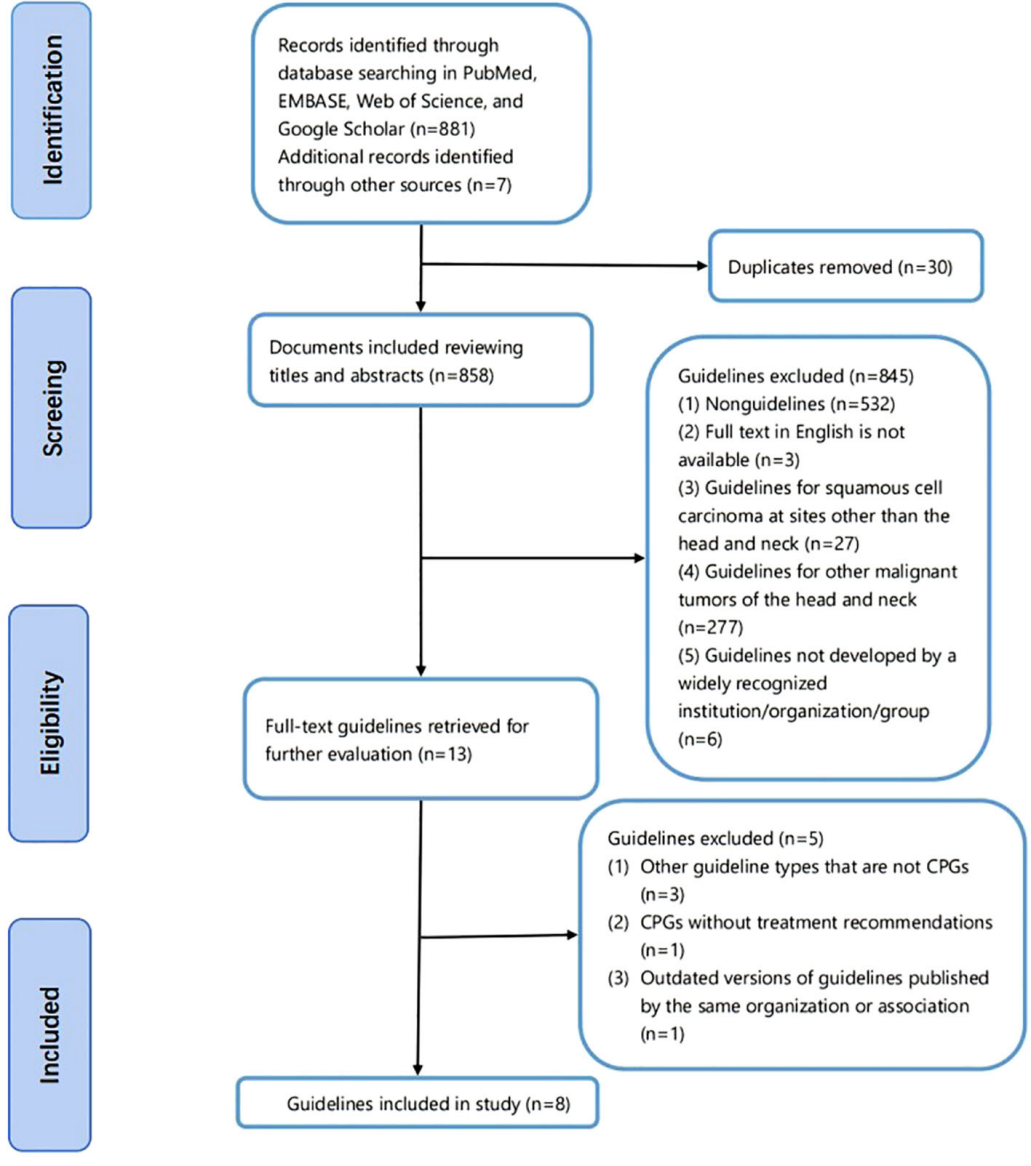
Flow diagram of the identification of guidelines.

A total of 881 documents were retrieved through the searches of the databases and websites of scientific societies and international associations, and since some guidelines are not be cataloged in traditional databases or published as scientific papers, we manually searched the websites of internationally recognized guideline publishers and obtained seven guidelines. The resulting literature was manually deduplicated to remove a total of 30 duplicates from multiple databases. Initial screening was performed by reading the titles and abstracts of the literature to assess whether the literature could be included in this study, and 845 documents were excluded based on the exclusion criteria. We obtained full-text of 13 guidelines, and eight guidelines that met the screening criteria were ultimately included (21–28). The screening process is shown in **Figure 4**. The eight CPGs included were jointly authored by multidisciplinary teams and belonged to the clinical practice guidelines proposed for squamous cell carcinoma of the head and neck; each of them provided clear recommendations, and the basic information of the 8 guidelines is shown in **Table 4**.

**Table 4:** Characteristics of included clinical practice guidelines.

Title	Year	First author	Organization	Abbreviation	Country	Funding	Tatget users	Evidence base	Guideline content
Reprint of “Squamous cell carcinoma of the oral cavity, larynx, oropharynx and hypopharynx: EHNS-ESMO-ESTRO Clinical Practice Guidelines for diagnosis, treatment and follow-up”	2020	J.-P. Machiels	European Head and Neck Society (EHNS), European Society for Medical Oncology (ESMO), European Society for Radiotherapy & Oncology (ESTRO)	EHNS,ESMO,ESTRO	Europe	ESOM	Clinicians	Expert consensus	Diagnosis, treatment and follow-up of Squamous cell carcinoma of the oral cavity, larynx, oropharynx and hypopharynx
SEOM clinical guidelines for the treatment of head and neck cancer	2020	R. Mesia	Spanish Society of Medical Oncology	SEOM	Spain	Merck. Dra	-	Expert consensus	Treatment of head and neck cancer
Management of the Neck in Squamous Cell Carcinoma of the Oral Cavity and Oropharynx: ASCO Clinical Practice Guideline	2019	Shlomo A. Koyfman, MD	American Society of Clinical Oncology	ASCO	USA	ASCO	Physicians and others	Expert consensus, systematic literature review	Management of the Neck in Squamous Cell Carcinoma of the Oral Cavity and Oropharynx
NCCN Clinical Practice Guidelines in Oncology (NCCN Guidelines) Head and Neck Cancers	2023	David G. Pfister	National Comprehensive Cancer Network	NCCN	USA	NCCN	Physician and other health care professionals	Expert consensus, systematic literature review	Treatment of head and neck cancer
Summary of Japanese clinical practice guidelines for head and neck cancer - 2022 update edited by the Japan society for head and neck cancer	2023	Akihiro Homma	Clinical Practice Guideline Committee of the Japan Society for Head and Neck Cancer	-	Japan	Japan Society of Head and Neck Cancer and the Ministry of Health, Labour and Welfare’s Comprehensive Research Project	Healthcare professionals, patients and their families	Expert consensus, systematic literature review	Review the latest evidence regarding head and neck cancer and to present the current standard approaches for diagnosis and treatment.
Guidelines for the Surgical Management of Oral Cancer: Korean Society of Thyroid-Head and Neck Surgery	2019	Young-Hoon Joo	Korean Society of Thyroid-Head and Neck Surgery Guideline Task Force	KSTHNS	Korean	-	Clinicians, patients, researchers, and health policy makers involved in diagnosis and treatment of oral cancer patients	Expert consensus, systematic literature review	Provide recommendations of diagnosis, work up, prevention, surgical treatment, postoperative care, and salvage surgery of oral cancer
A Quality Initiative of the Program in Evidence-Based Care (PEBC), Cancer Care Ontario (CCO)Systemic Therapy in the Curative Treatment of Head and Neck Squamous Cell Cancer	2022	Winquist E	The Expert Panel on Systemic Therapy in Head and Neck Squamous Cell Cancer	CCO/PEBC	Canada/Ontario	-	Clinicians and other healthcare professionals involved in the management of LASCCHN.	Expert consensus, systematic literature review	Make recommendations regarding treatment strategies for cure and/or organ preservation in patients with locally advanced nonmetastatic squamous cell carcinoma of the head and neck
Oral cavity cancer: diagnosis, treatment and follow-up	2014	VINCENT GRÉGOIRE	Belgian Health Care Knowledge Centre	KCE	Belgium	INAMI - RIZIV	All care providers involved in the management of patients with oral cavity squamous cell cancer	Expert consensus, systematic literature review	provides recommendations for the staging, treatment, follow-up and supportive care of patients with oral cavity squamous cell cancer

### 3.2 Results of the consistency tests

The intragroup correlation coefficients for the final evaluation results by the two evaluators are shown in **Tables 5**–**7**. The intragroup agreement between the two reviewers was good in all 16 domains of the three scales, which suggests that the opinions of the two reviewers were consistent, to a large extent, during the review process.

**Table 5:** Intraclass correlation coefficients for inter-rater reliability across the 6 AGREE II domains.

AGREE II						
	Domain 1 Scope and purpose	Domain 2 Stakeholder involvement	Domain 3 Rigor of development	Domain4 Clarity of presentation	Domain 5 Applicability	Domain 6 Editorial independence
Intraclass correlation coefficient (ICC)	0.79	0.85	0.94	0.80	0.83	0.82
95% Confidence interval	[0.56,0.90]	[0.68,0.93]	[0.90,0.96]	[0.58,0.91]	[0.69,0.91]	[0.56,0.93]

**Table 6:** Intraclass correlation coefficients for inter-rater reliability across the 3 AGREE-REX domains.

AGREE-REX			
	Domain 1 Clinical applicability	Domain 2 Values and preferences	Domain 3 Implementability
Intraclass correlation coefficient (ICC)	0.77	0.87	0.85
95% Confidence interval	[0.54,0.89]	[0.71,0.94]	[0.60,0.95]

**Table 7:** Intraclass correlation coefficients for inter-rater reliability across the 7 RIGHT domains.

RIGHT							
	Domain 1 Basic information	Domain 2 Background	Domain 3 Evidence	Domain 4 Recommendations	Domain 5 Review and quality assurance	Domain 6 Funding and declaration and management of interests	Domain 7 Other information
Intraclass correlation coefficient (ICC)	0.90	0.79	0.75	0.87	0.83	0.96	0.97
95% Confidence interval	[0.83, 0.94]	[0.67, 0.86]	[0.56,0.87]	[0.79, 0.92]	[0.54,0.94]	[0.92,0.98]	[0.92,0.99]

### 3.3 Quality of methodology

The two independent reviewers calculated the scores for each of the six AGREE II domains after scoring the eight guidelines, and the specific results are shown in **Table 1**. The eight selected guidelines generally performed better in domain 1, “Scope and purpose”, and domain 4, “Clarity of presentation”, and worse in domain 5, “Applicability”, and domain 3, “Rigor of development”. The highest score was for domain 4, “Clarity of presentation”, at 92.4% ± 11.4%, followed by domain 1, “Scope and purpose”, at 81.3% ± 13.9%. The lowest score was for domain 5, “Application”, at 61.5% ± 25.7%, followed by domain 3, “Rigor of development”, at 70.6% ± 24.5%. The guidelines authored by the ASCO, CCO, NCCN, and KCE scored ≥60% in five or more domains and were considered to be high quality, while the rest of the guidelines were of average quality. At the same time, there was a small difference in the scores for domains 1 and 4 and a large difference in the scores for domains 5 and 6.

From the perspective of a single guideline, the highest scores among the eight guidelines selected were assigned to the ASCO guideline, with an average score of 99.5%. The lowest scoring guideline was developed by the Korean Thyroid-Head and Neck Surgery Working Group, with an average score of 57.3%, with lower scores for domain 5, “Applicability”; domain 6, “Editorial independence”; and domain 2, “Stakeholder involvement”. Lower scores were the main reason for the overall low scores of some guidelines. The lowest variabilities in scores were observed for the guidelines generated by the ASCO and KCE, while the highest variabilities in the NCCN and ESOM scores were related to large differences in their scores for different domains.

### 3.4 Quality of recommendations

**Table 2** summarizes the ratings of the eight guidelines in the three AGREE-REX domains. Domain 1, “Clinical applicability,” had the highest score of 77.4% ± 15.7%, whereas domain 2, “Values and preferences,” had the lowest score of 37.8% ± 29.7%. The AGREE-REX scale scores were generally relatively low compared with those of the AGREE II scale. From a single-guideline perspective, the guidelines developed by the ASCO continued to receive the highest scores, while the guidelines developed by the ESOM received the lowest scores. All of the guidelines produced by the ASCO and KCE had domain scores ≥60%, whereas no guidelines produced by the ESOM and SEOM had domain scores ≥60%.

### 3.5 Quality of reporting

The scores for each domain of the RIGHT checklist are shown in **Table 3**, and among the eight selected guidelines, the overall performance was better for domains 1 and 2 and worse for domains 6 and 5. The highest scoring domain was domain 1, “Basic Information,” with 95.3% ± 4.1%, followed by domain 2, “Background,” with 76.6% ± 13.7%; the lowest scoring domain was domain 6, “Funding and declaration and management of interests,” with a score of 43.8% ± 34.5%, followed by domain 5, “Review and quality assurance”, with a score of 57.8% ± 35.3%. At the individual guideline level, the highest average scores were assigned to the guidelines authored by the ASCO, while the lowest scores were assigned to the guidelines authored by the NCCN. Of the eight guidelines, four had a mean score ≤60%.

## 4 Discussion

### 4.1 Innovativeness

Clinical practice guidelines have been increasingly used in the clinical care process, influencing patient diagnosis, treatment, care and outcomes; at the same time, there is a high demand from guideline users for trusted health advice (29). Although most guidelines are developed based on expert consensus and systematic literature searches, the lack of uniform standards for their preparation may affect their applicability and dissemination to the extent that it may be difficult to achieve the objectives of the developers. In previous studies, researchers have mostly used a single AGREE II tool to evaluate the methodological quality of guidelines, ignoring their reporting quality and clinical applicability. In this study, we used three tools, AGREE II, AGREE-REX, and RIGHT, to evaluate the methodology, quality of recommendations, and quality of reporting for eight clinical practice guidelines in three dimensions and analyzed the reasons while deriving more standardized guidelines through comparison. The results of this study provide methodological and reporting references, which could effectively improve future guideline development.

### 4.2 Methodological quality of the CPGs for HNSCC

Of the eight guidelines that were systematically evaluated, those produced by the ASCO, CCO, NCCN, and KCE were rated as “high quality” on the AGREE II scale, suggesting that these four guidelines may be of high methodological quality. The ASCO guidelines performed well in each domain, with no apparent weaknesses, while the CCO and KCE guidelines scored relatively low in domain 5(66.7% and 89.6%, respectively), “Applicability,” indicating that they lacked descriptions of the facilitators and barriers to guideline application, were not comprehensive enough to provide advice and/or tools for applying their recommendations, failed to consider clinical resources that may be needed to apply recommendations, and did not provide high-quality surveillance and monitoring tools. The lack of clinical resources required and the lack of clarity about the criteria for surveillance/audits imply that the guidelines do not adequately account for the different contexts of clinical application, which should clearly articulate how facilitators and impediments influence the guideline development process and the development of recommendations. There is a clear gap between the “independence” of the NCCN guidelines in domain 6 (scored 12.5%) and its performance in other domains, suggesting that the formulation of guideline recommendations fails to reflect a research process that is free of interference from other domains and interests, such as politics and economics. The potential for conflicts of interest and bias among members of guideline development groups has been demonstrated (30, 31), which may affect the impartiality of the guideline content.

Overall, the included guidelines performed well in domain 1, “Scope and purpose”, and domain 4, “Clarity of presentation”. High scores in the “Clarity of presentation” domain indicate that the recommendations communicated by the guidelines to the target group are clear and easy to recognize and understand, while high scores in “Scope and purpose” indicate that the guideline developers have a clear plan and vision for the purpose of the guidelines.

At the same time, these guidelines scored low in domain 5, “Applicability”, and domain 3, “Rigor of development”, for example, The KOREAN guideline scored 22.9% in domain 5 and the EHNS/ESMO/ESTRO guideline scored 32.2% in domain 3. The applicability of any CPG depends on several factors, such as rigorous development, clear presentation, editorial independence, adequate dissemination, and adequate implementation strategies (32). “Applicability” mainly affects the application and dissemination of the guidelines, whose main goal is to guide clinical practice in HNSCC treatment and to ensure clinical efficacy. which requires not only the preparation of well-established guidelines based on rigorous scientific evidence but also their practical use and dissemination by physicians at the clinical level. Increased “applicability” has been shown to be effective in improving adherence to the guideline in clinical practice (33, 34). “Rigor” is the most important and comprehensive area of clinical practice guideline development, encompassing 8 of the 23 items, and how recommendations are formulated based on the evidence will have a direct impact on the clinical applicability of the guideline, a process that largely determines the quality of CPGs (35)(e.g. The EHNS/ESMO/ESTRO guideline scored 32.2% and the SEOM guideline scored 50.0% in domain 3). In this study, we found that the lack of rigor was mainly due to the lack of a clear description of the criteria, strengths, and limitations of evidence selection in some guidelines, as well as to the lack of external review before publication or to the failure to clearly reflect the review process in the guidelines. We observed that only some of the selected guidelines provided complete and detailed descriptions of the search strategy and the grading of the level of evidence through appropriate tools such as GRADE, which ensured the transparency of the evidence search process while avoiding potential bias (36–38). Well-established and rigorous guidelines should clearly articulate the process of recommendation formulation and the sources of evidence (39, 40), as it has been shown (41–43) that an increase in the level of evidence is associated with an increase in the specificity of guideline recommendations and that specific guidelines provide clinicians with more feasible recommendations.

### 4.3 Quality of recommendations in the CPGs for HNSCC

Recommendations in high-quality guidelines should be evidence based, applicable, implementable, and take into account the values of all stakeholders. Among the included guidelines, the ASCO and KCE guidelines scored high and had no significant areas of weakness, with each guideline scoring more than 60% in each area, which we considered to be a high-quality guideline. Meanwhile, the guidelines prepared by the ESOM and SEOM did not score more than 60% in any area; therefore, we considered these two guidelines to have a low quality of recommendations at the quality of the recommendations level.

In domain 1, “Clinical applicability”, six guidelines received high overall scores, indicating that evidence, target user applicability, and patient/population applicability were adequately considered in the guideline development process, while the two guidelines written by the ESOM and SEOM scored lower. The problems of the ESOM guidelines centered on Item 2, “Target User Applicability”, where recommendations may be less likely to be adhered to due to a lack of adequate consideration of the applicability to the practice setting of the target user in formulating the recommendation, while the guideline written by SEOM scored lower in Item 1, “Evidence”, which is related to a lack of consideration of the practice setting of the target user during the guideline writing process. This is related to the lack of a thorough review of the quality of evidence during the guideline development process, which leads to a lack of evidence transparency and may hinder discussion and communication among guideline developers (44).

In general, domain 2, “Values and preferences”, was poorly represented, with all but two guidelines, namely, the ASCO and KCE guidelines, scoring less than 30% in this domain; in particular, the values and preferences of policy-makers and guideline developers were not adequately described, suggesting that during the guideline development process, guideline developers disregarded the values and preferences of multilevel stakeholders, which may result in guideline recommendations that are biased toward health professionals to the extent that they are not meaningful to patients (45–47). On the other hand, writing guidelines that do not take into account the values of the target populations and policy-makers may result in guideline adherence and outcomes that fall short of expectations, with high-quality studies not being used as they should be and a waste of resources (48, 49).

In domain 3, “Implementability”, most of the guidelines, with the exception of the ASCO and KCE guidelines, performed poorly. We noted that most of the guidelines lacked recommendations that were specific to different settings, such as a region and resource allocation, and that guideline developers did not tailor their recommendations to target populations from different settings, especially resource-poor settings, which may reduce the adherence to the guideline recommendations. For clinical practice guidelines to have an impact on the course of treatment and, ultimately, on outcomes, it is necessary to ensure that they are actually implemented and used by the final clinician in accordance with the guideline recommendations (50), and their “implementability” will be a determining factor in the ultimate adoption of guideline recommendations in the clinic.

The above analysis shows that quality control of recommendations is still a common problem in the development of CPGs for HNSCC, which requires attention of guideline developers.

### 4.4 Reporting quality of the CPGs for HNSCC

**Figure 5:**
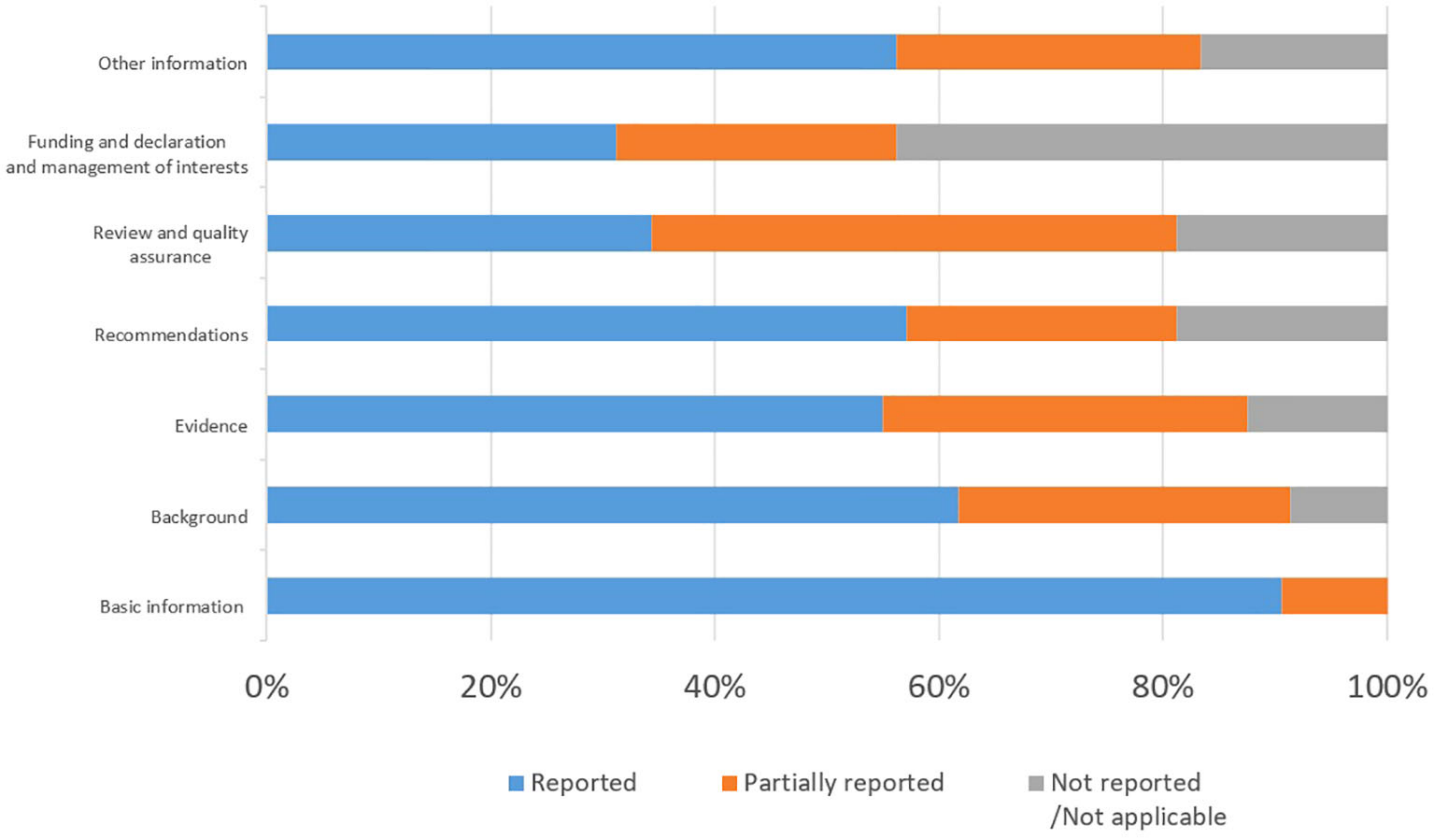
Reporting rates to each domain of the RIGHT checklist.

Of the eight guidelines included, only the ASCO and KCE guidelines received high reporting quality scores, with the average reporting rate for each domain being greater than 70%. The reporting rates to each domain of the RIGHT checklist see **Figure 5**.

Generally, with the exception of domain 1, “Basic Information”, the quality of reporting in all domains was less than optimal. The main reason for the poor quality of guideline reporting is the lack of awareness of standardization during the development process, which may lead to the loss of clinical guidance (51). In domain 5, “Review and quality assurance”, most of the guidelines provided partial information, i.e., only stated that the guideline had been externally reviewed but did not describe the specific review process, consideration of review comments, results, and quality control procedures, which should be strengthened by reporting on the review process and improving the quality of reporting in this domain. This finding is also consistent with the AGREE II requirements for quality of evidence.

Domain 6, “Funding and declaration and management of interests,” was underreported, particularly in Item 19a, “Describe the types of conflicts of interest relevant to the development of the guidance,” and Item 19b, “Describe methods for evaluating and managing conflicts of interest”. This may be due to the fact that most guidelines simply state that no conflict of interest exists without analyzing the type of conflict and response in detail. This result suggests that the current CPGs for HNSCC lack sufficient standardization and transparency in terms of external review and financial conflicts of interest, which may be related to factors such as space limitations by journals, the lack of awareness among guideline developers about the standardization of guideline reporting, the lack of explicit reporting of the corresponding content at the time of writing, and conflicts of interest that may influence the interpretation and recommendations for the treatment of disease (52, 53). Moreover, the lack of awareness of the guidelines and the shortcomings of guidelines in this area are reflected in other guidelines on different topics (54–56). By analyzing the content of specific entries, we found that “Accreditation and Quality Assurance” and “Declaration and Management of Funding and Conflicts of Interest” were the most important changes to the AGREE II areas of “rigor” and “independence”. The “independence” domain (11), and thus the quality of reporting in the development of normative guidelines, is another layer of complementing the quality of methodology.

### 4.5 Summary

In the present study, we observed that although most of the guidelines (except the CCO guideline) adopted the GRADE system to grade the quality of evidence, there still were more recommendations based on expert consensus in general, and multicenter randomized controlled trials were not sufficiently represented as a source of recommendations, which may lead to a greater lack of specificity, concreteness, and persuasiveness of recommendations. At the same time, the failure of some guidelines to clearly describe the process of evidence retrieval, selection criteria, and other processes of recommendation formation may lead to some controversy about the rationality and rigor of the process of recommendation formation.

Furthermore, we found that some guidelines that are recognized in the field of HNSCC treatment as important guides for clinical treatment approaches (e.g., the NCCN guideline and the ESMO guideline) did not have a significant advantage in scoring after evaluation with the three guideline evaluation tools. This may be related to the fact that the current guideline evaluation tools do not assess the clinical guidance content of the guidelines themselves and do not judge the validity of the recommendations but only evaluate their methodological and reporting quality. This result indicates, on the one hand, the lack of rigor in the process of evidence retrieval, selection criteria, and the formulation of recommendations for current guidelines with important clinical guidance, which may lead to disagreement among guideline users in the process of clinical application; on the other hand, the scores of the evaluation tool do not represent the true clinical value of the guidelines, to a certain extent, which may lead to the selection of guideline recommendations based on the evaluation results of the evaluation tool. Therefore, in the process of guideline development, clinical experts should fully cooperate with the methodology research group and emphasize the methodological and reporting quality of the guidelines.

Finally, guidelines are also of great importance in guiding the development of health policies, not only by providing clinicians with recommendations to help them base their practice on scientific evidence but also by improving the use of existing health-care resources. Therefore, guidelines should have a standardized development process and be able to provide sufficiently effective recommendations to guide decision-makers in the development and implementation of health policies. It is worth noting that most of guidelines worldwide were developed in high-income countries and regions because the compilation of CPGs requires a large investment of time and human, financial, and other resources that may be difficult for low- and middle-income countries to afford; therefore, low- and middle-income countries may need to consider the applicability of international guidelines and whether it is feasible to implement them in the context of the local economic level and cultural background.

### 4.6 Limitations

This study has several limitations as follows: (1) the number of guidelines included in this study was small, possibly because of the lack of comprehensiveness in the databases searched and the fact that some guidelines were not included in the databases, which led to the exclusion of some other available CPGs; furthermore, there is a risk of individual bias when manually searching for guidelines in the guideline repositories of associations and organizations; (2) CPGs that could not be published in full text in English were excluded from the study, and the language barrier was another limitation of the lack of comprehensiveness of the guidelines included in this study. (3) the evaluation with the three guideline evaluation tools used qualitative evaluation scales, which may have been influenced by reviewer subjectivity; (4) although scoring the quality of guidelines by counting the number of domains with scores ≥60% is a well-recognized and widely used methodology, it has not yet been formally validated; and (5) our study only assessed the methodology of the guidelines, the quality of the recommendations, and the quality of the reports and did not evaluate their impacts on clinical practice or patient outcomes.

## 5 Conclusions

The quality of most current clinical practice guidelines on the diagnosis and treatment of squamous cell carcinoma of the head and neck is relatively average, and there is still a need for guideline developers to further improve the quality of their guidelines according to the guideline development specifications, preferably with the involvement of methodology professionals, to enhance the applicability and implementability of the guidelines. According to the results of the comprehensive evaluation using the AGREE II tool, four of the eight group-written guidelines demonstrated high-quality content; according to the results of the AGREE-REX evaluation, only two guidelines met the specifications; according to the results of the RIGHT inventory evaluation, the quality of reporting was good for four guidelines, of which the ASCO guidelines performed excellent in the evaluation across the three tools. Therefore, it is recommended that the squamous cell carcinoma of the head and neck-related guidelines written by the ASCO be used for reference by health care professionals and patients. In conclusion, the methodological quality, recommendations and reporting quality of the current CPGs on head and neck squamous cell carcinoma still need to be improved, and in the process of guideline development in the future, it is recommended that guideline developers consider basing their work on the three evaluation tools as a framework to improve the quality of the current guidelines on head and neck squamous cell carcinoma to better guide clinical practice. Ultimately, the objective of guideline development is to provide more effective guidance for clinical practice.

## Data Availability

The original contributions presented in the study are included in the article/supplementary material. Further inquiries can be directed to the corresponding authors.
